# The nuclear variant of bone morphogenetic protein 2 (nBMP2) is expressed in macrophages and alters calcium response

**DOI:** 10.1038/s41598-018-37329-5

**Published:** 2019-01-30

**Authors:** Claudia M. Tellez Freitas, Haley R. Burrell, Jonard C. Valdoz, Garrett J. Hamblin, Carlee M. Raymond, Tyler D. Cox, Deborah K. Johnson, Joshua L. Andersen, K. Scott Weber, Laura C. Bridgewater

**Affiliations:** 1 Department of Microbiology and Molecular Biology, Brigham Young University, Provo, Utah United States of America; 2 Department of Chemistry and Biochemistry, Brigham Young University, Provo, Utah United States of America

## Abstract

We previously identified a nuclear variant of bone morphogenetic protein 2 (BMP2), named nBMP2, that is translated from an alternative start codon. Decreased nuclear localization of nBMP2 in the nBmp2NLS^tm^ mouse model leads to muscular, neurological, and immune phenotypes—all of which are consistent with aberrant intracellular calcium (Ca^2+^) response. Ca^2+^ response in these mice, however, has yet to be measured directly. Because a prior study suggested impairment of macrophage function in nBmp2NLS^tm^ mutant mice, bone marrow derived (BMD) macrophages and splenic macrophages were isolated from wild type and nBmp2NLS^tm^ mutant mice. Immunocytochemistry revealed that nuclei of both BMD and splenic macrophages from wild type mice contain nBMP2, while the protein is decreased in nuclei of nBmp2NLS^tm^ mutant macrophages. Live-cell Ca^2+^ imaging and engulfment assays revealed that Ca^2+^ response and phagocytosis in response to bacterial supernatant are similar in BMD macrophages isolated from naïve (uninfected) nBmp2NLS^tm^ mutant mice and wild type mice, but are deficient in splenic macrophages isolated from mutant mice after secondary systemic infection with *Staphylococcus aureus*, suggesting progressive impairment as macrophages respond to infection. This direct evidence of impaired Ca^2+^ handling in nBMP2 mutant macrophages supports the hypothesis that nBMP2 plays a role in Ca^2+^ response.

## Introduction

Our group has reported the existence of a nuclear variant of the growth factor bone morphogenetic protein 2 (BMP2), designated nBMP2^1^. This variant protein is produced by translation from an alternative downstream start codon that eliminates the N-terminal endoplasmic reticulum signal peptide, thus preventing the protein’s delivery to the secretory pathway. Instead, nBMP2 is translated in the cytoplasm and translocated to the nucleus by means of an embedded bipartite nuclear localization signal (NLS)^1^. Using immunohistochemistry, we have detected nBMP2 in skeletal muscle nuclei and in the nuclei of CA1 neurons in the hippocampus^2,3^.

To examine the function of nBMP2, we generated a mutant mouse strain (nBmp2NLS^tm^) in which a three-amino acid substitution in the NLS inhibits translocation of nBMP2 to the nucleus while still allowing normal synthesis and secretion of the conventional BMP2 growth factor^2^. The mice appear overtly normal and are fertile. They do, however, lack nBMP2 in myonuclei, and electrophysiological studies revealed that skeletal muscle relaxation is significantly slowed after stimulated twitch contraction, a process that is regulated by intracellular Ca^2+^ transport. Consistent with impaired intracellular Ca^2+^ transport, sarco/endoplasmic reticulum Ca^2+^ ATPase (SERCA) activity is decreased in skeletal muscle^2^. The mutant mice also lack nBMP2 in CA1 hippocampal neurons, and electrophysiological studies revealed reduced long-term potentiation (LTP) in the hippocampus^3^. LTP is dependent on intracellular Ca^2+^ transport and is thought to be the cellular equivalent of learning and memory^4–6^. Behavioral tests revealed that the nBMP2 mutant mice have impaired object recognition memory^3^.

Intracellular Ca^2+^ elevation also regulates the activation and differentiation of several different types of immune cells including T cells, B cells, dendritic cells, and macrophages^7–10^. To see if nBmp2NLS^tm^ mutants had compromised immune response, mice were challenged by systemic infection with *Staphylococcus aureus*. While the mutants’ immune response to a primary infection appeared normal, their immune response to a secondary infection challenge 30 days later resulted in higher levels of bacteremia, increased mortality, and failure of spleens to enlarge normally^11^. Although we did not observed differences in the total number of macrophages in spleen, thymus, or lymph node from wild type compared to mutant mice, we did observe that after the secondary infection, spleen from nBmp2NLS^tm^ mutant mice showed fewer hemosiderin-laden macrophages than spleen from wild type mice^11^. Macrophages in the spleen accumulate hemosiderin by phagocytosing damaged red blood cells and hemoglobin, which would be present in the blood stream of *S. aureus*-challenged mice due to the hemolysins that *S. aureus* expresses^12–14^. The observation of fewer hemosiderin-laden macrophages in the spleens of mutant mice after a secondary infection suggested to us that macrophage phagocytic activity might be impaired in the absence of nBMP2, potentially providing us with an accessible cell type in which to directly test our hypothesis that intracellular Ca^2+^ response is disrupted in the absence of nBMP2.

To interrogate if nBMP2 might play a role in Ca^2+^ response, we isolated macrophages from wild type and nBmp2NLS^tm^ mutant mice. These macrophages included bone marrow derived (BMD) macrophages from uninfected mice, and splenic macrophages from mice that had undergone primary and secondary infections with *S. aureus*^15^. Live-cell Ca^2+^ imaging as well as bead engulfment assays were performed to measure intracellular Ca^2+^ response and phagocytic activity. These analyses revealed deficient Ca^2+^ response and phagocytosis in splenic macrophages isolated from mutant mice after secondary systemic infection with *S. aureus*, but not in BMD macrophages from naïve mice, suggesting that as nBmp2NLS^tm^ mutant cells respond to infection over time, Ca^2+^ response is progressively impaired.

## Results

### The nuclear variant nBMP2 is expressed in BMD and splenic macrophages from wild type mice

**Figure 1:**
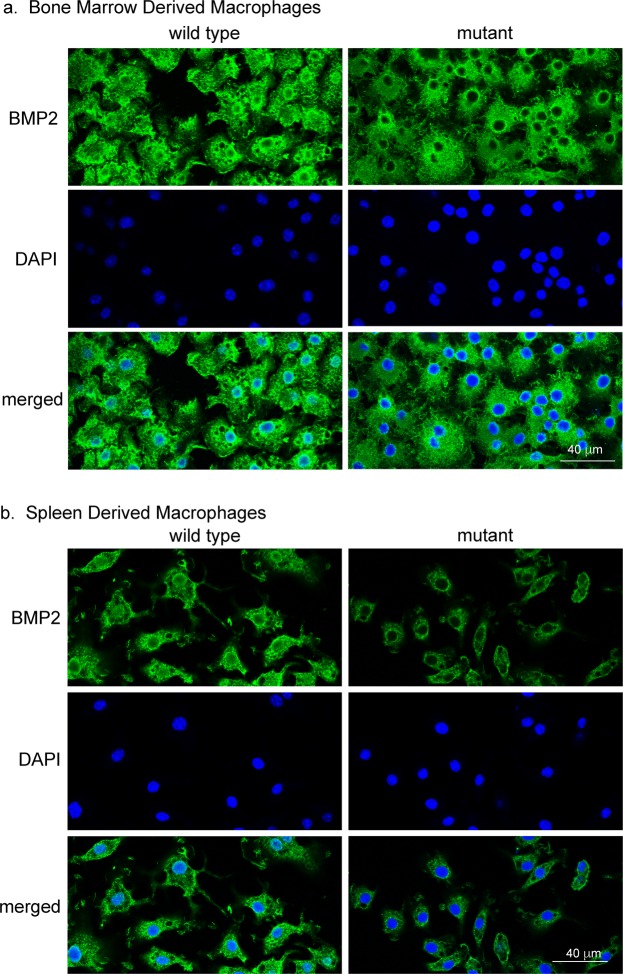
BMD macrophages and splenic macrophages express nBMP2, which is decreased in the nuclei of nBmp2NLS^tm^ mutant macrophages. (**a**) BMD macrophages and (**b**) splenic macrophages were stained with anti-BMP2 antibody (green) and counterstained with DAPI (blue), demonstrating that nBMP2 is expressed and localized to the nucleus in wild type macrophages, and that nuclear translocation of nBMP2 is inhibited in mutant macrophages. BMP2 labeling within the cytoplasm is present in both wild type and mutant cells as expected, because the targeted mutation allows translation of nBMP2 in the cytoplasm but inhibits nuclear translocation, and it allows normal synthesis and secretion of conventional BMP2.

**Figure 2:**
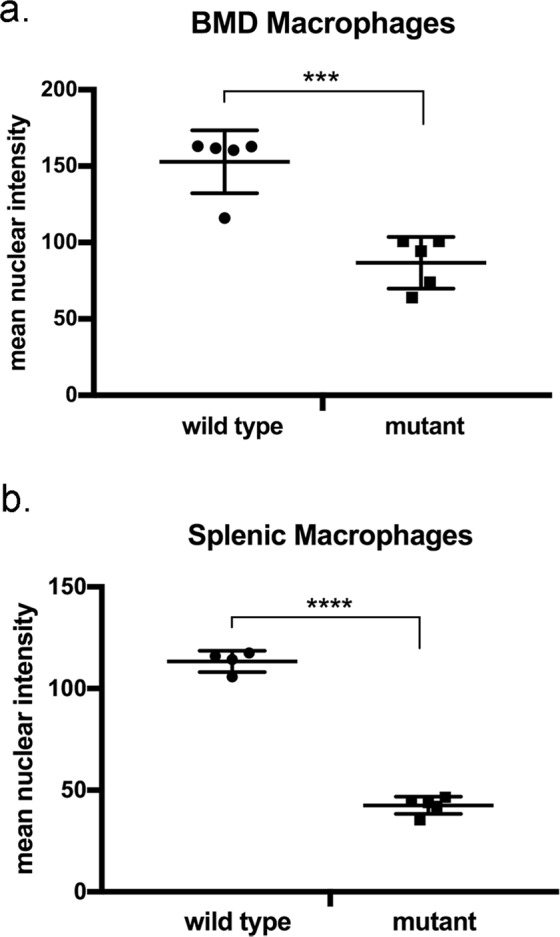
Quantification of nBMP2 nuclear staining intensity. Five images each were analyzed for wild type and mutant BMD macrophages and for mutant splenic macrophages. Four images were analyzed for wild type splenic macrophages. Each image contained between 10 and 93 cells, and the number of cells analyzed per group ranged from 100 to 337. ImageJ was used to outline DAPI-stained regions and quantify BMP2 immunostaining as the sum of pixel intensities within each nucleus. The mean density of BMP2 immunostaining was then calculated for all nuclei in an image. An unpaired, two-tailed t-test was performed to compare nuclear staining between wild type and mutant cells. For BMD wild type vs. mutant macrophages, p = 0.0005. For splenic wild type vs mutant macrophages, p < 0.0001.

To determine whether nBMP2 is expressed in macrophages, BMD macrophages and splenic macrophages were isolated from naïve (uninfected) wild type and nBmp2NLS^tm^ mutant mice and differentiated *in vitro*, and immunocytochemistry was performed using an anti-BMP2 antibody that binds to both BMP2 and nBMP2. Consistent with our prior observation of impaired immune response in nBmp2NLS^tm^ mutant mice^11^, nBMP2 was detected in the nuclei of wild type BMD (Fig. 1a) and splenic (Fig. 1b) macrophages. As expected, nBMP2 was significantly decreased in macrophage nuclei from nBmp2NLS^tm^ mutant mice (Fig. 1a,b, mutant). ImageJ software quantification of immunofluorescence images showed that the density of nuclear BMP2 staining was significantly more intense in wild type compared to mutant macrophages in both BMD macrophages (p = 0.0005) and splenic macrophages (p < 0.0001) (Fig. 2). BMP2 staining was visible throughout the cytoplasm of both wild type and mutant macrophages, as expected, given that nBMP2 is synthesized in the cytosol before being translocated to the nucleus and that the conventional BMP2 growth factor is synthesized in the rough ER and translocated through the Golgi before being secreted from the cell.

### BMD macrophages from uninfected nBmp2NLS^tm^ mutant mice and wild type mice have similar Ca^2+^ response

**Figure 3:**
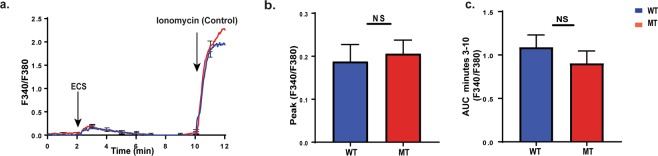
Naïve bone marrow derived (BMD) macrophages from nBmp2NLS^tm^ mutant mice and wild type mice have a similar Ca^2+^ response. Naïve BMD macrophages from wild type (WT) and nBmp2NLS^tm^ mutant (MT) mice were loaded with Fura-2AM for live-cell Ca^2+^ imaging. During imaging, cells were stimulated at 2 min with *E. coli* supernatant (ECS), then at 10 min with ionomycin as a positive control. (**a**) Average curves showing intracellular Ca^2+^ response in wild type and nBmp2NLS^tm^ mutant BMD macrophages. Fluorescence ratios (F340/F380) were measured at 3 sec intervals from 0–12 min (n = 38 cells). Error bars (s.e.m.) are shown at one min intervals. (**b**) Average (±s.e.m.) of peak Ca^2+^ influx (F340/F380) in wild type and nBmp2NLS^tm^ mutant BMD macrophages (n = 38 cells). (**c**) Area under the curve (AUC) of F340/F380 ratios from minutes 3 to 10 min shows sustained intracellular Ca^2+^ levels (n = 38 cells). NS, not significant.

Naïve BMD macrophages isolated from femurs and tibias of uninfected mice were differentiated and activated *in vitro* then plated for live-cell Ca^2+^ imaging. Plated cells were loaded with Fura-2AM, a UV-excitable ratiometric calcium indicator that changes its excitation in response to Ca^2+^ binding; Fura-2AM emits at 380 nm when Ca^2+^ is not bound, and at 340 nm when Ca^2+^ binds to the dye. The fluorescence ratio (F340/F380), increases as cytosolic Ca^2+^ levels increase^16^. At the 2 min time point, supernatant from *Escherichia coli* (ECS) cultures was added to stimulate Ca^2+^ flux (Fig. 3a)^17–19^. Following this stimulation, there were no observable differences between naïve mutant and wild type BMD macrophages in peak Ca^2+^ response (Fig. 3b) or sustained Ca^2+^ levels (Fig. 3c).

### Splenic macrophages isolated from nBmp2NLS^tm^ mutant mice after secondary infection show impaired Ca^2+^ response

In our prior study, immune deficiencies in nBMP2NLS^tm^ mice were detectable only after the mice received a secondary infection with *S. aureus*^11^. Because our current experiments revealed no significant differences in Ca^2+^ response in naïve BMD macrophages from mutant compared to wild type mice, we decided to replicate the *in vivo* conditions of our previous work by examining splenic macrophage harvested from mice after a secondary infection with *S. aureus*, and by using *S. aureus* supernatant as the stimulus to trigger Ca^2+^ flux^11^. Although *S. aureus* is a gram positive bacteria that does not produce LPS, it does produce liphoteichoic acid (LTA), which is similarly able to activate macrophages^20,21^. Thirty-five days after primary systemic *S. aureus* infections, mice were given a second injection of *S. aureus*, and splenic macrophages were isolated 3 days later.

**Figure 4:**
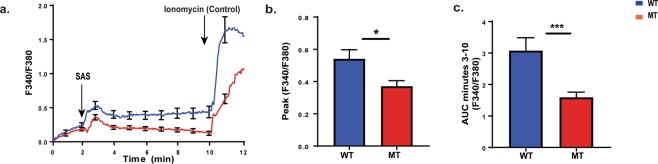
Splenic macrophages collected from nBmp2NLS^tm^ mutant mice after secondary infection have an impaired Ca^2+^ response. Splenic macrophages from wild type (WT) and nBmp2NLS^tm^ mutant (MT) mice were loaded with Fura-2AM for live-cell Ca^2+^ imaging. During imaging, cells were stimulated at 2 min with *S. aureus* supernatant (SAS), then at 10 min with ionomycin as a positive control. (**a**) Average curves showing intracellular Ca^2+^ response in wild type and nBmp2NLS^tm^ mutant splenic macrophages. Fluorescence ratios (F340/F380) were measured at 3 sec intervals from 0-12 min (n = 44 cells). Error bars (s.e.m.) are shown at one min intervals. (**b**) Average ± s.e.m. of peak Ca^2+^ influx (F340/F380) in wild type and nBmp2NLS^tm^ mutant splenic macrophages shows a significant difference (n = 44 cells). (**c**) AUC of F340/F380 ratios from minutes 3 to 10 min shows a significant difference in sustained intracellular Ca^2+^ levels (n = 44 cells). *p < 0.05, **p < 0.01, ***p < 0.0001.

After one week *in vitro* maturation, splenic macrophages were loaded with Fura-2AM for live-cell Ca^2+^ imaging experiments. *S. aureus* supernatant (SAS) was used to stimulate Ca^2+^ flux at the 2-min time point (Fig. 4a). Compared to the lack of a difference in naïve BMD macrophages, it is particularly striking that peak Ca^2+^ response was significantly decreased (p = 0.0335) in mutant splenic macrophages after secondary infection (Fig. 4b). Sustained Ca^2+^ levels as measured by the area under the curve (AUC) from minutes 3–10 was also significantly decreased (p = 0.0008) (Fig. 4c).

### BMD macrophages from uninfected nBmp2NLS^tm^ mutant mice and wild type mice show similar phagocytic activity

**Figure 5:**
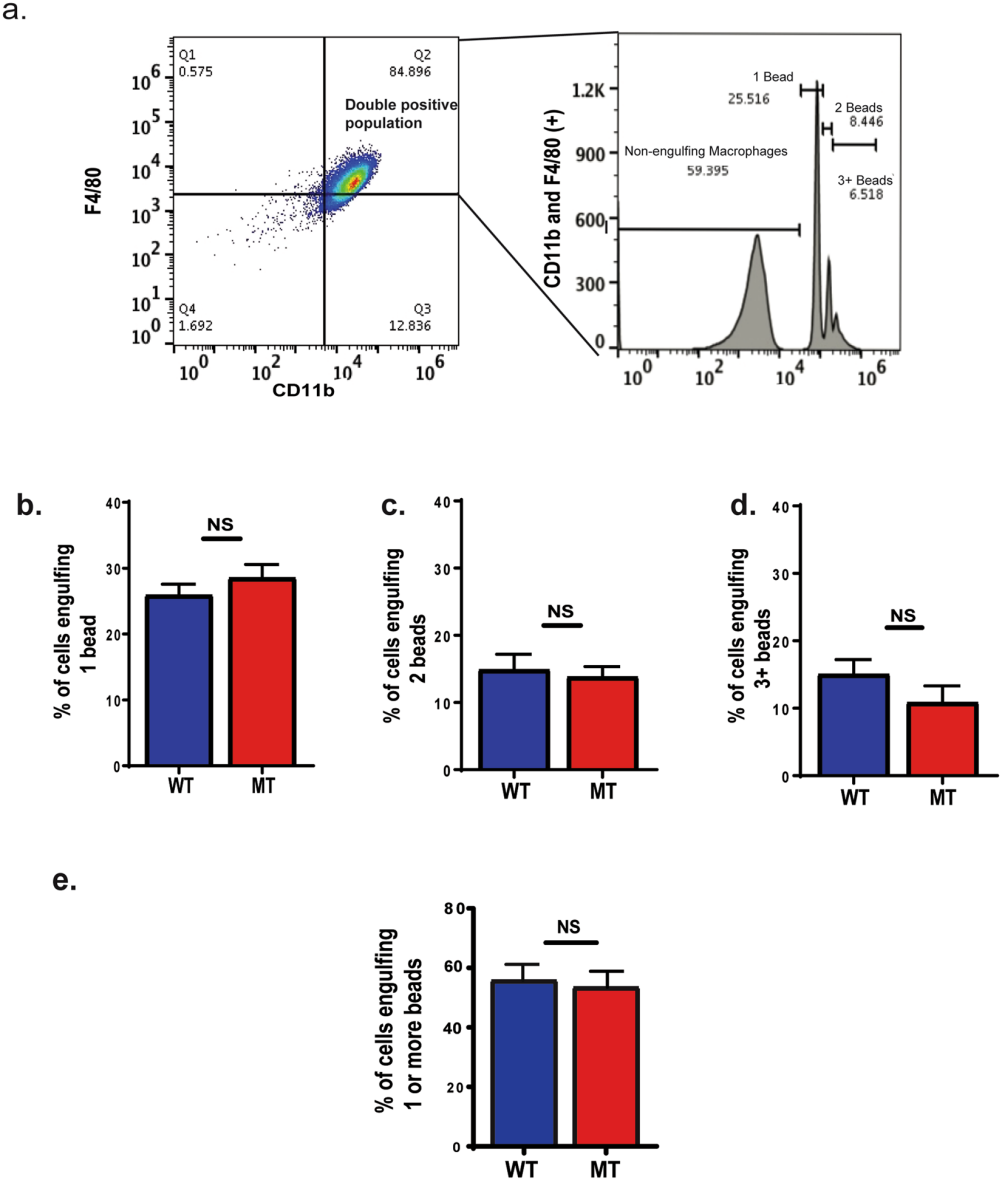
Naïve bone marrow derived (BMD) macrophages from nBmp2NLS^tm^ mutant mice and wild type mice show similar phagocytic activity. After incubation with fluorescent microspheres, macrophages were analyzed by flow cytometry. (**a**) A representative analysis is shown. The F4/80 and CD11b double positive population was selected, and from this gate a histogram was produced to identify macrophages that had engulfed 1, 2, or 3 or more beads. The percentages of total double positive cells represented within each peak are indicated. (**b**) Percent of cells engulfing 1 bead, (**c**) percent of cells engulfing 2 beads, and (**d**) percent of cells engulfing 3 or more beads. (**e**) Percent of cells engulfing one or more beads. N = 3 pairs of wild type and 3 pairs of mutant mice. NS, not significant.

To test phagocytic activity of naïve BMD macrophages (meaning macrophages that were isolated from uninfected mice) from nBmp2NLS^tm^ mutant compared to wild type mice, we measured fluorescent bead engulfment by CD11b and F4/80 positive cells with flow cytometry (Fig. 5a)^22–28^. We observed no differences in the phagocytic activity of naïve BMD macrophages from nBmp2NLS^tm^ mutant compared to wild type mice (Fig. 5b–e).

### Splenic macrophages from nBmp2NLS^tm^ mutant mice show impaired phagocytic activity

**Figure 6:**
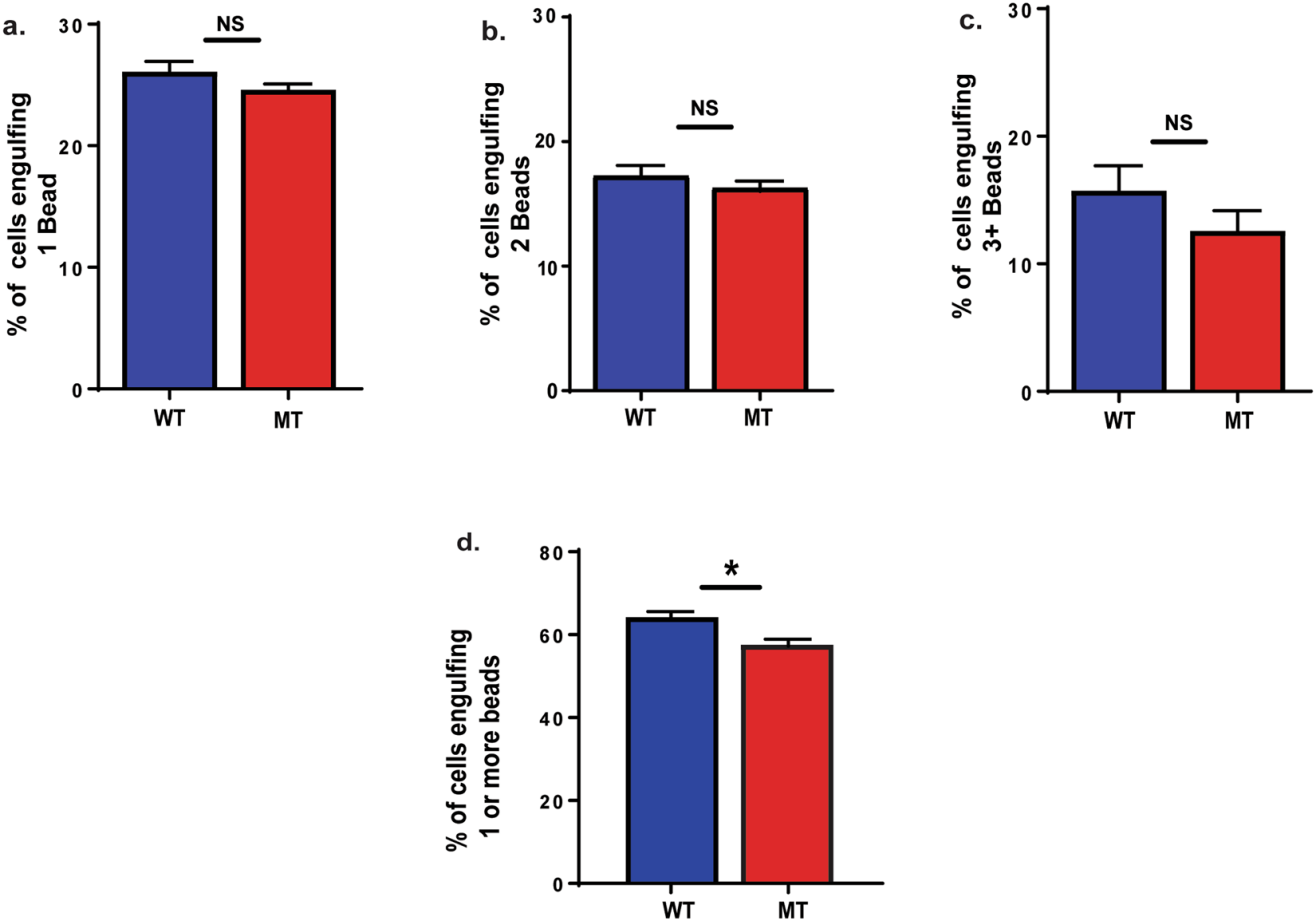
Splenic macrophages collected from nBmp2NLS^tm^ mutant mice after secondary infection show impaired engulfment activity. After incubation with fluorescent microspheres, macrophages were analyzed by flow cytometry as described in Fig. 3. (**a**) Percent of cells engulfing 1 bead, (**b**) percent of cells engulfing 2 beads and, (**c**) percent of cells engulfing 3 or more beads. (**d**) Percent of cells engulfing one or more beads. N = 3 pairs of wild type and 3 pairs of mutant mice. NS, not significant. *p < 0.05.

To test phagocytic activity in macrophages isolated from mice after secondary infection, splenic macrophages were isolated from wild type and nBmp2NLS^tm^ mutant mice 3 days after mice received a second systemic infection with *S. aureus*, and fluorescent bead engulfment was measured as described above. While differences between wild type and mutant macrophages did not reach significance when subgroups that engulfed 1, 2, or 3 or more beads were analyzed individually (Fig. 6a–c), there was a significant reduction in overall mutant phagocytic activity (p = 0.0176) when the subgroups were pooled (Fig. 6d). These data suggest a possible relationship between the decreased Ca^2+^ response and reduced phagocytosis in nBmp2NLS^tm^ mutant splenic macrophages.

## Discussion

The role of BMP2 in macrophages is unknown and remains an area of active research. BMP2 has been reported to be constitutively expressed in M1 (inflammatory) macrophages^29^. Other studies have shown that BMP2 expression is upregulated as macrophages shift toward the pro-healing/anti-inflammatory M2 phenotype^30,31^. BMP2 secretion by macrophages promotes migration of vascular smooth muscle cells, and macrophages in the intestinal muscularis secrete BMP2 to signal enteric neurons^32,33^. Reports of BMP2 expression by hematopoietic cells, in particular macrophages, are relevant to this study because nBMP2 can be produced from the same mRNA as the conventional secreted BMP2 growth factor—any time BMP2 mRNA or BMP2 growth factor is detected, the potential for nBMP2 synthesis exists^1^. Accordingly, we have demonstrated by immunofluorescence that both BMD macrophages and splenic macrophages express the nuclear variant of BMP2, nBMP2, and that nBMP2 is decreased in the nuclei of macrophages from nBmp2NLS^tm^ mutant mice.

Previously, we demonstrated that deficiency of nBMP2 in the nucleus impairs secondary immune response as evidenced by diminished spleen enlargement, poor clearance of *S. aureus* from the bloodstream, and increased mortality after secondary infection^11^. We have also shown that deficiency of nBMP2 in myonuclei is correlated with slowed skeletal muscle relaxation after contraction, and deficiency of nBMP2 in the nuclei of hippocampal neurons is correlated with learning/memory deficits^2,3^. Each of these phenotypes is consistent with deficiencies in intracellular Ca^2+^ transport, but until now, no direct measurements of intracellular Ca^2+^ have been performed in cells from nBmp2NLS^tm^ mutant mice. The discovery that macrophages express nBMP2 (Fig. 1) provided an accessible cell type in which to directly address the question of whether nBMP2 plays a role in intracellular Ca^2+^ response.

We found that intracellular Ca^2+^ response was impaired in mutant splenic macrophages after secondary infection with *S. aureus*, but not in mutant BMD macrophages isolated from uninfected mice, even though both macrophage types expressed nBMP2. Recent work has revealed that innate immune cells can undergo memory-like adaptive responses to increasing pathogen load, and the deficient Ca^2+^ response in splenic macrophages after secondary infection might represent a failure of those adaptive responses^34,35^. Alternatively, it may be that the effects of nBMP2 deficiency in the nucleus are simply cumulative, causing a Ca^2+^-handling phenotype that becomes progressively more severe as cells differentiate and mature. A progressive phenotype is consistent with our previously reported observation that hippocampal long-term potentiation (LTP) was normal in 3-week-old nBmp2NLS^tm^ mutant mice but deficient in 3-month-old mice^3^. Progressive impairment of intracellular Ca^2+^ response has received attention recently as a potential mechanism for both brain and muscle aging^36–38^, suggesting that nBMP2 dysfunction could contribute to premature aging or aging-related diseases.

Deficiency of nBMP2 in the nucleus also produced a significant decrease in the total phagocytic activity of splenic macrophages from nBmp2NLS^tm^ mutant mice, suggesting that mutant cells may be less effective at clearing pathogens from the blood stream. This is consistent with prior studies suggesting that intracellular Ca^2+^ mobilization plays a role in macrophage phagocytic activity. For example, impaired Ca^2+^ response in macrophages from Trpm4(−/−) mutant mice led to decreased phagocytic activity, resulting in bacterial overgrowth and translocation to the bloodstream^39^. Intracellular Ca^2+^ levels increase during Fcɣ receptor (FcR)-mediated phagocytosis^40–42^, and the loss of CaMKK2, a calcium-dependent kinase, left macrophages unable to phagocytose bacteria or synthesize cytokines in response to bacterial lipopolysaccharide (LPS)^43^.

Although evidence supports the involvement of Ca^2+^ response in macrophage phagocytic activity, the scale of the decreased phagocytosis by splenic macrophages observed in our study seems insufficient to account for the markedly increased mortality of nBmp2NLS^tm^ mutant mice after secondary infection^3^. We cannot rule out the possibility that the bead engulfment assay did not fully reflect the severity of phagocytosis impairment in splenic macrophages. Liver macrophages also play a role in bacterial clearance, and it is possible that the absence of nBMP2 in the nucleus affects their function more severely^44,45^. In addition, the absence of nBMP2 in the nucleus might affect other immune system cell types besides macrophages, and it is possible that another cell type, or perhaps several cell types together, account for the increased mortality of nBmp2NLS^tm^ mutant mice after secondary infection^3^. Indeed, BMP2 (and therefore potentially nBMP2) is expressed by a specialized endothelial population in the early embryo, termed hemogenic endothelium, that gives rise to hematopoietic stem cells^46^. The absence of nBMP2 at the earliest stages of hemogenesis could therefore impact a wide range of immune cell types. BMP2 is also expressed in human cord blood cells, including those that express CD34, a hematopoietic progenitor cell antigen^47^, and acute bleeding triggers upregulation of BMP2 expression in hematopoietic stem cells^48^. BMP2 expression is also found in mature B cells, where it is upregulated in response to infection with *Aggregatibacter actinomycetemcomitans*^49^. It is possible, therefore, that nBMP2 impacts the activation or function of other immune cell types in addition to macrophages, and the combined functional deficits account for the increased mortality in nBmp2NLS^tm^ mutant mice after secondary infection.

It will be important, in future work, to elucidate the molecular mechanisms underlying the Ca^2+^ response differences between macrophages from wild type and nBMP2 mutant mice. Differences may stem from impaired uptake or release of Ca^2+^ from endoplasmic reticulum stores, as suggested by the decreased SERCA activity observed in skeletal muscle of nBMP2 mutant mice^2^. Alternatively, transport of Ca^2+^ could be impaired at the macrophage cell membrane, consistent with observations that increasing extracellular Ca^2+^ levels can improve phagocytosis^50,51^. Neurons and muscle cells are excitable cells and are therefore equipped with a different set of ion channels and transporters than are macrophages, and so it will be important to examine molecular details of the Ca^2+^ handling defect in all three cell types. This work has thus opened the way for future studies into the molecular interactions and activities of nBMP2.

Questions about how nBMP2 functions from inside the nucleus to affect Ca^2+^ response also remain to be answered. The novel protein nBMP2 was first identified from among nuclear proteins that had been isolated using DNA affinity chromatography, but subsequent experiments failed to show direct binding of nBMP2 to DNA, and the amino acid sequence of nBMP2 contains no predicted DNA-binding domain^1^. It is possible that nBMP2 interacts indirectly with DNA through a transcription factor, and future studies of nBMP2’s impact on the expression of genes involved in Ca^2+^ signaling will be informative.

In summary, this study supports our working hypothesis that aberrant intracellular Ca^2+^ response is the mechanism that unites the otherwise disparate muscle, neurological, and immune phenotypes observed in nBmp2NLS^tm^ mutant mice^2,3,11,52–54^. In doing so, this study has paved the way for future work to elucidate the precise molecular nature of the Ca^2+^ signaling disruptions in nBMP2 mutant cells and to understand how nBMP2’s interactions in the nucleus impact Ca^2+^ signaling.

## Materials and Methods

### Research Animals

This study was carried out in strict accordance with recommendations in the Guide for the Care and Use of Laboratory Animals^55^. The protocol was approved by the Institutional Animal Care and Use Committee (IACUC) of Brigham Young University (protocol numbers 15-0107 and 15-0603).

Mice were housed in a temperature-controlled (21–22 °C) room with a 12:12 hr light-dark cycle and fed standard rodent chow and water *ad libitum*. The nBmp2NLS^tm^ mice were constructed on a Bl6/129 background, as described^2^. The homozygous wild type and mutant mice used in this study were obtained by breeding heterozygotes, and genotyping was performed as previously described^3^. All experiments were performed with male mice at least 6 months of age.

### BMD and Splenic Macrophage Isolation

BMD macrophages were obtained from femurs and tibias of wild type and nBmp2NLS^tm^ mutant mice and were differentiated in culture at 37 °C with 5% CO_2_ for 7 days in macrophage medium (DMEM (HyClone), 10% fetal bovine serum (FBS) (HyClone), 20% supernatant from L929 mouse fibroblast as a source of macrophage colony-stimulating factor (M-CSF), 5% heat inactivated horse serum (Sigma), 1 mM sodium pyruvate (Gibco by Life Technologies), 1.5 mM L-glutamine (Thermofisher), 10 u/ml penicillin, 10 μg/ml streptomycin (Gibco by Life Technologies)) prior to plating for immunocytochemistry, Ca^2+^ imaging or engulfment assays.

Spleens from wild type and nBmp2NLS^tm^ mutant mice were homogenized in phosphate buffered saline (PBS). The homogenate was filtered, pelleted at 450 × g for 5 min, suspended in lysis buffer (155 mM NH_2_Cl, 10 mM KHCO_3_, 0.1 mM EDTA) on ice for 3–5 min to lyse erythrocytes, and then washed with 37 °C macrophage media and plated in macrophage medium in 6-well plates. After 3 days of culture at 37 °C in 5% CO_2_, medium was replaced to remove non-adherent cells^56^. On day 4, 100 ng/ml lipopolysaccharide (LPS) was added to the culture medium to stimulate differentiation, and cells were incubated for 3–4 more days^57^. Differentiated cells were then plated for immunocytochemistry, Ca^2+^ imaging, or engulfment assays.

### Immunocytochemistry

Immunocytochemistry was performed using BMD and splenic macrophages. Following macrophage isolation and 7-day differentiation as described above, cells were plated on coverslips that were pre-treated with 0.025% HCl in PBS for 20 min to facilitate cell attachment. Cells were cultured for 1–2 days to reach 70–90% confluence, then fixed at 37 °C in 4% paraformaldehyde for 10 min. Epitopes were exposed through antigen retrieval using 5% sodium citrate and 0.25% Tween-20 in ddH2O, pH 6.0, at 95 °C for 10 min. Cells were permeabilized using 0.1% Triton X-100 then blocked for 1.5 hr at room temperature (RT) using SEA BLOCK blocking buffer (ThermoFisher Scientific, 37527). The samples were then probed with 1:50 anti-BMP2 antibody (Novus Biologicals, NBP1-19751) diluted in 10% SEA BLOCK blocking buffer in 0.1% Tween-20/PBS (PBS-T), overnight at 4 °C. The probed slides were then stained with anti-rabbit Alexa Fluor 488 (ThermoFisher Scientific, A-11034) for 1 hr at RT. Afterwards, nuclei were stained by incubating the slides in 1:5000 DAPI in PBS-T for 15 min., then slides were mounted using Prolong^TM^ Gold Antifade Mountant (Life Technologies, P10144) and cured overnight prior to microscopic imaging. Cells were imaged using a Leica TCS-SP8 confocal microscope with 63X magnification, using the same laser intensities for all samples. Appropriate laser lines were used such as 405 nm for DAPI and 488 nm for BMP2-Alexa Fluor 488.

Comparison of nuclear BMP2 staining intensity between wild type and mutant cells was performed on tiff versions of confocal microscope images using ImageJ to create tracings of DAPI-stained regions and to calculate the mean pixel intensity of nBMP2 staining within each nucleus. Mean nuclear staining intensity was calculated for each image, and groups were compared using an unpaired, two-tailed t-test in GraphPad Prism.

### *S. aureus* Bacterial Infections

*S. aureus* ATCC strain 12600 was cultured in tryptic soy broth liquid culture alternating with standard streak plating on mannitol salt agar (Thermo Fisher Scientific) for counting. To prepare bacteria for injections, 100 µl of overnight liquid culture was transferred into a new 15 ml broth culture and grown until OD_600_ reached 1.0, then pelleted and resuspended in 15 ml of PBS with 20% glycerol, aliquoted, and stored at −80 °C for 3 weeks before injection. Frozen stock concentration was verified one day before the infection by thawing a single aliquot and performing standard serial dilution plate counts. On the day of infection, *S. aureus* was diluted from the frozen stock to the desired concentration in PBS, and mice received a 200 μl retroorbital injection using a 1 ml syringe and 27-gauge needle. The injected volume contained a priming dose of 1 × 10^4^ CFU/g body weight on day 0 (primary infection), and a dose of 3 × 10^5^ CFU/g body weight on day 35 (secondary infection). Macrophages were harvested three days later.

### Bacterial Supernatant Preparation

Bacterial supernatant obtained from *E. coli* K12 and *S. aureus* 12600 was used to stimulate Ca^2+^ fluxes in BMD and splenic macrophages^19,58^. A single colony was picked from an agar plate and inoculated into liquid broth overnight culture. The next day, 1 ml of the overnight culture was inoculated into 15 ml liquid broth and incubated with shaking at 37 °C until culture reach an OD_600_ of 1–1.3. Cells were then pelleted by centrifugation at 1,800 × g for 12 min at 4 °C, and supernatant was collected.

### Calcium Imaging

BMD and splenic macrophages were isolated and differentiated in culture for 7 days as described above, then seeded on 8-chambered coverglasses (Nunc 155411, Thermo Scientific) and incubated overnight in macrophage medium at 37 °C in 5% CO_2_. For BMD macrophages, 10 ng/ml LPS from E. coli O55:B5 (Sigma) was included in the overnight incubation to activate cells. The next day, cells were loaded with 3 μM Fura-2AM (Invitrogen) in Ringers solution containing Ca^2+^ to be used as an extracellular source during the Ca^2+^ imaging assay (150 mM NaCl, 10 mM glucose, 5 mM HEPES, 5 mM KCl, 1 mM MgCl_2_, 2 mM CaCl_2_, pH 7.4) for 30 min at 37 °C in 5% CO_2_, washed with Ringers solution, then incubated for another 30 minutes at 37 °C in Ringers solution. Calcium imaging was performed at room temperature using an Olympus IX51 inverted microscope equipped with a xenon arc lamp. Fura-2AM loaded macrophages were excited using 340 nm and 380 nm excitation filters, and images of 340 nm, 380 nm, and transmitted light were capture using a florescence microscope camera (Q Imaging Exi Blue) with a 20x objective (N.A. 0.75) at 3-sec intervals. At the 2-min time point in each imaging protocol, 20 μl of bacterial supernatant was added to stimulate Ca^2+^ flux. Ionomycin (1 μM final concentration) was added at the 10-min time point as a positive control. 10–20 representative cells were selected as regions of interest in each frame, and F340:F380 ratios were calculated and analyzed using CellSens software from Olympus. Each individual cell’s fluorescence was normalized to its first recorded value according to the equation (F-Fo)/Fo, where F is the fluorescence at the specific time point, and Fo is the fluorescence value at time 0^19,59^.

### Engulfment Assay

BMD and splenic macrophages were isolated and differentiated in culture for 7 days as described above, then seeded in 12-well culture plates for flow cytometry-based engulfment assays^22–28^. 100% FBS was used to resuspend 2.0 μm phycoerythrin-conjugated polychromatic red latex microspheres (Polysciences, Inc.) to prevent beads from sticking to the cell membranes during engulfment^23^. The ~10^9^ particles/ml concentration was chosen to ensure that beads were not a limiting factor in phagocytosis rates^23^. Macrophages were then activated by adding LPS from *E. coli* O55:B5 (Sigma) to a final concentration of 10 ng/ml and incubated for 1 hour at 37 °C and 5% CO_2_. Media was removed and cells were rinsed with cold PBS, then collected and analyzed by flow cytometry using an Attune flow cytometer (Applied Biosystems by Life technologies). Cells were pre-treated with anti-CD16/32 antibodies (14-0161-85 eBioscience) to prevent non-specific antibody binding, then surface stained with APC-conjugated anti-CD11b antibodies (17-0112-82 eBioscience) and FITC-conjugated anti-F4/80 antibodies (11-4801-82 eBioscience). Doublets were removed based on forward scatter width (FSC-W)/forward scatter area (FSC-A), and the F4/80 and CD11b double positive population was selected. From within this gate, engulfing macrophages were distinguished from non-engulfing macrophages based on phycoerythrin fluorescence, and macrophages could be further distinguished based on the engulfment of one, two, or three or more beads. Results were analyzed using FlowJo software (Tree Star).

### Data Analysis

All assays were performed as at least three independent repeats, each in triplicate. Area under the curve (AUC) was determined using GraphPad Prism. Statistical significance was assessed using unpaired two-tailed Students T test in GraphPad Prism.

## Data Availability

All data generated or analyzed during this study are included in this published article. Biological reagents will be made available on request.
